# Predicted Archaic 3D Genome Organization Reveals Genes Related to Head and Spinal Cord Separating Modern from Archaic Humans

**DOI:** 10.3390/cells9010048

**Published:** 2019-12-24

**Authors:** Daniel Batyrev, Elisheva Lapid, Liran Carmel, Eran Meshorer

**Affiliations:** 1 Department of Genetics, The Alexander Silberman Institute of Life Sciences, The Hebrew University of Jerusalem, Edmond J. Safra Campus, Givat Ram, Jerusalem 9190401, Israel; daniel.batyrev@mail.huji.ac.il (D.B.); eli7lapid@gmail.com (E.L.); 2 The Edmond and Lily Safra Center for Brain Sciences (ELSC), The Hebrew University of Jerusalem, Edmond J. Safra Campus, Givat Ram, Jerusalem 9190401, Israel; 3 The Rachel and Selim Benin School of Computer Science and Engineering, The Hebrew University of Jerusalem, Edmond J. Safra Campus, Givat Ram, Jerusalem 9190401, Israel

**Keywords:** ancient DNA, epigenetics, DNA methylation, genome organization, gene regulation, archaic humans, comparative epigenomics

## Abstract

High coverage sequences of archaic humans enabled the reconstruction of their DNA methylation patterns. This allowed comparing gene regulation between human groups, and linking such regulatory changes to phenotypic differences. In a previous work, a detailed comparison of DNA methylation in modern humans, archaic humans, and chimpanzees revealed 873 modern human-derived differentially methylated regions (DMRs). To understand the regulatory implications of these DMRs, we defined differentially methylated genes (DMGs) as genes that harbor DMRs in their promoter or gene body. While most of the modern human-derived DMRs could be linked to DMGs, many others remained unassigned. Here, we used information on 3D genome organization to link ~70 out of the remaining 288 unassigned DMRs to genes. Combined with the previously identified DMGs, we reinforce the enrichment of these genes with vocal and facial anatomy, and additionally find significant enrichment with the spinal column, chin, hair, and scalp. These results reveal the importance of 3D genomic organization in understanding gene regulation by DNA methylation.

## 1. Introduction

DNA methylation of cytosine residues is a key epigenetic mechanism in mammals [1]. Almost all scattered cytosines within CpG dinucleotides are methylated in human cells. In contrast, regions known as CpG islands, which contain relatively dense clusters of CpGs are often protected from DNA methylation. CpG islands are mostly found in promoter regions of many house-keeping and other highly expressed genes. Once methylated, the corresponding genes become inactivated. In addition, a genome-wide survey of DNA methylation of enhancers in healthy and cancerous cell types revealed a strong association between enhancer methylation and suppression of expression of the corresponding gene [2]. In addition to its fundamental role in regulating gene expression, DNA methylation also regulates imprinting and the silencing of transposable elements and other repetitive and low-complexity regions of the genome. Aberrant DNA methylation is associated with diseases including many types of cancer. Therefore, DNA methylation contains a crucial layer of information on genome activity, function, and phenotype [3].

As the interplay between DNA methylation and transcription regulation may work through modulating the effects of remote elements such as enhancers, in order to link specific enhancers with their target genes, the higher order 3D structure of the genome should be considered [4]. The human genome is partitioned into local domains formed by genomic loops, of which around 10,000 have currently been identified. These loops frequently link promoters and enhancers, correlate with gene expression, or can act as insulators for gene regulation [5]. Interestingly, such higher-order domains show a very high degree of conservation across cell types and species. Loop anchors typically occur at domain boundaries and can bind the CCCTC-binding factor (CTCF) insulator protein [6,7].

In an evolutionary context, DNA methylation might help explain some of the molecular basis for phenotypic diversity, as it is widely accepted that many phenotypic differences between closely related species are attributed to changes in gene regulation [8]. Nevertheless, the evolution of the human methylome and the processes driving such changes are poorly understood [9].

Recently, we developed a novel method to infer genome-wide pre-mortem DNA methylation patterns from archaic genomes, opening the possibility of studying the methylomes of extinct species. Using this method, we identified thousands of differentially methylated regions (DMRs) separating humans, Neanderthals, and Denisovans [10,11]. Of those, 873 are modern human-derived DMRs (MH-derived DMRs), where the methylation change occurred along the modern human lineage after the split from Neanderthals and Denisovans. Five hundred and eighty-five of these human-derived DMRs are associated with 520 unique MH-derived differentially methylated genes (DMGs), defined as genes whose body or promoter overlap with MH-derived DMRs. Using Gene ORGANizer, a tool that links genes to body parts that they phenotypically affect [11], we reported that the list of MH-derived DMGs is significantly enriched with genes affecting vocal and facial anatomy [10].

Here, we attempted to link some of the remaining 288 MH-derived DMRs to protein-coding genes [12], based on the 3D organization of the human genome. We used 3D genomic information from 4 different cell lines to obtain a consensus higher-order organization. We then analyzed our DMR data in the context of the consensus map to obtain potential long-distance interactions between genes and DMRs. Using this method, we were able to assign additional 70 MH-derived DMRs to genes, reporting 69 new DMGs. We further show that these DMGs not only strengthen the association of methylation changes with the anatomy of the voice and face, but also highlight additional body parts which are significantly enriched with DMGs, including the chin and spinal column.

## 2. Materials and Methods

In order to identify topologically associating domains (TADs) preserved between four different cell types, we downloaded Hi-C data for Human Mammary Epithelial Cells (HMEC), Human Umbilical Vein Endothelial Cells (HUVEC), Human fetal lung cells (IMR90), and Normal Human Epidermal Keratinocytes (NHEK) cell types [6]. We chose IMR90 as the basis cell type, having the highest number of identified TADs (Supplementary Table S1). For each TAD in the IMR90 cell type, we calculated a score between 0 and 1 representing the tendency of this domain to be also present in the other cell types. A score of 1 is obtained when a TAD appears in the exact same coordinates in all cell types, whereas a score of 0 is obtained when a TAD does not appear in any of the other cell types. To compute the score, let us represent the IMR90 cell type by subscript A, and the other three cell types by subscripts B, C, and D. Let $T_{X}\;$ be the set of all TADs in cell type X. Then, for every TAD in IMR90, $T_{i}\in T_{A}\;$, the score would be:

(1) $$S_{i}=\mathrm{median}\left.\left(\underset{T_{j}\in T_{B}}{\max}\frac{T_{i}\cap T_{j}}{T_{i}\cup T_{j}},\underset{T_{k}\in T_{C}}{\max}\frac{T_{i}\cap T_{k}}{T_{i}\cup T_{k}},\underset{T_{l}\in T_{D}}{\max}\frac{T_{i}\cap T_{l}}{T_{i}\cup T_{l}}\right.\right).$$

Here, a TAD is viewed as an interval and ∩ and ∪ represent the length of the intersection or union of the intervals of two TADs.

In order to identify TADs that are conserved across all cell types, we performed a permutation test, where we replaced each $T_{i}\in T_{A}$ (with score $S_{i}>0.5$) by a random interval of the same length. We used these data to recompute scores for all TADs, which yielded a *p*-value per TAD. The corresponding adjusted *q*-values were then computed using FDR.

## 3. Results

### 3.1. Identifying Conserved Topologically Associating Domains (TADs)

**Figure 1:**
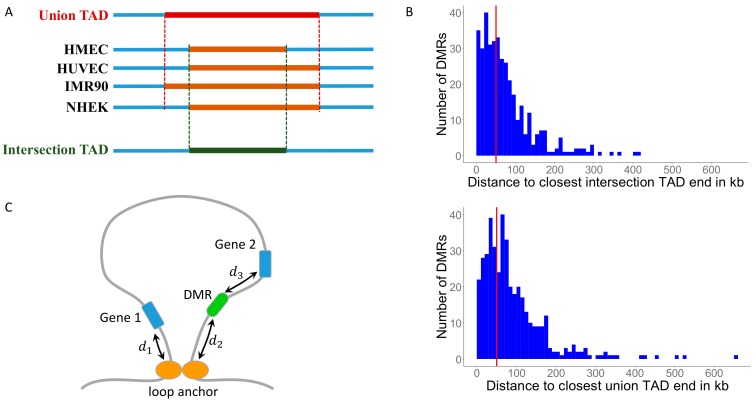
(**A**) Definition of a conserved Topologically Associating Domain (TAD) boundaries, as either the intersection of all intervals, or as their union. (**B**) Histograms of the distances between Differentially Methylated Regions (DMRs) inside TADs and their closest TAD boundary in intersection-TADs and union-TAD, respectively. Red vertical line represents the 50 kb cut-off value. (**C**) Definitions of spatial and linear distances between a DMR and a gene. The spatial distance of the DMR from gene 1 is $d_{1}+d_{2}$. The linear distance of the DMR from gene 2 is $d_{3}$.

In order to study methylation changes in the human skeletal system in the context of 3D genomic architecture, we needed data on topologically associating domains (TADs) in modern human bones. As TAD maps in human bone are not available, we used data from four diverse non-bone cell types, and generated consensus TAD maps (see Materials and Methods), assuming that TADs that are conserved across these cell types are likely to appear in bone as well. TADs are highly conserved both between different cell types as well as between different species: of the 3331 domains annotated in mouse CH12-LX cells (murine lymphoma), 1649 (50%) are orthologous to domains in the human lymphoblastoid cells GM12878 [6]. In addition, the four cell lines we used in our analysis include epithelial cells (HMEC), endothelial cells (HUVEC), fetal lung fibroblasts (IMR90), and epidermal keratinocytes (NHEK), representing all three embryonic germ layers. Therefore, a consensus TAD map of these cell lines would, in all probability, represent TADs that are very likely to be found in bone as well. The data were produced by the same laboratory, minimizing experimental variations [6]. As the location of the TAD boundaries (called anchors) in these experiments cannot be determined precisely, conserved TADs might display different boundaries in different cell types. We have therefore defined the boundaries of conserved TADs in two ways: (1) the TAD interval is taken as the section that is shared across all cell types (hereinafter denoted intersection-TADs), and (2) the TAD interval is taken as the section that is the union of all the intervals in the different cell types (hereinafter denoted union-TADs) (Figure 1A). We therefore defined highly conserved TADs as those TADs that meet the following three criteria: i) A minimum TAD length of 100 kb, ii) Their conservation score $S_{i}$ (see Materials and Methods) satisfies $S_{i}>0.5,$ and iii) Adjusted *q*-value in permutation test < 0.05 (see Materials and Methods).

Overall, we identified 2902 conserved TADs, which are 37.8% of the total number of TADs detected in IMR90. The intersection-TADs and union-TADs span 28% and 32% of the modern human genome, respectively.

### 3.2. MH-Derived DMRs Tend to Reside Inside TADs

In order to study the spatial organization of the DMRs with respect to the consensus TADs, we first tested whether MH-derived DMRs tend to reside, at least partially, within conserved TADs. To this end, we used a Monte Carlo simulation, randomly allocating DMRs while keeping their length, GC content, and CpG density similar. Using 200 simulations, we found that for both intersection-TADs and union-TADs the fraction of DMRs that reside inside TADs is much higher than expected by chance (*p* < 0.005, Table S2). From the simulations we have also computed the expected fraction of DMRs inside TADs, which we compared to the observed number using a χ^2^-test (*p* < 10^−12^, Table S2). Together, these analyses show that DMRs tend to reside inside conserved TADs much more than expected by chance.

### 3.3. MH-Derived DMRs Inside TADs Tend to Reside Closer to the Conserved TAD Ends

Loop domains are thought to affect gene regulation by bringing remote regulatory elements to the proximity of their targets if both reside inside the TAD and near the loop anchor. Therefore, we wanted to test if MH-derived DMRs inside a TAD are closer to the TAD boundary than expected by chance. For this, we defined a DMR residing up to 50 kb from a TAD boundary as proximal to the boundary, as enhancers are typically closer than 50 kb to their target [11]. Using a Monte Carlo simulation with 10,000 repetitions, we found that MH-derived DMRs are indeed much closer to TAD boundaries than expected by chance (*p* < 10^−4^, Table S3). A similar conclusion was obtained using a χ^2^-test on the expected value of the simulations (*p* = 1.2 × 10^−54^ and *p* = 1.4 × 10^−58^ for intersection- and union-TADs, respectively, Table S3). Together, these results suggest that MH-derived DMRs reside within 50 kb of a TAD boundary much more frequently than expected by chance (Figure 1B).

### 3.4. Mapping DMRs to Genes

The 288 MH-derived DMRs that do not reside in a promoter or a gene body, and hence could not be linked to genes based on their genomic location, are hereinafter called non-genic DMRs (ngDMRs). To test if 3D genomic organization data may help in linking ngDMRs to genes, we redefined distances between a DMR and a gene in a way that accounts for topological information. For each pair of MH-derived ngDMR and a gene, we calculated two distances: a linear distance, measured directly along the DNA strand, but computed only if the ngDMR and the gene reside in the same TAD; and a spatial distance that is computed when the DMR and the gene are in different ends of the same TAD (Figure 1C). We then associated ngDMRs with the closest gene if the distance between them (either linear or spatial) was smaller than 50 kb.

We performed this procedure for both intersection-TADs and union-TADs. For intersection-TADs we found 70 ngDMRs that could be linked to 69 DMGs. Of them, 61 are new DMGs, and 8 already appeared in our previous list, as they also overlap DMRs in their body or promoter. Using union-TADs, we could link 69 ngDMRs with 69 DMGs, of which 61 are new.

**Figure 2:**
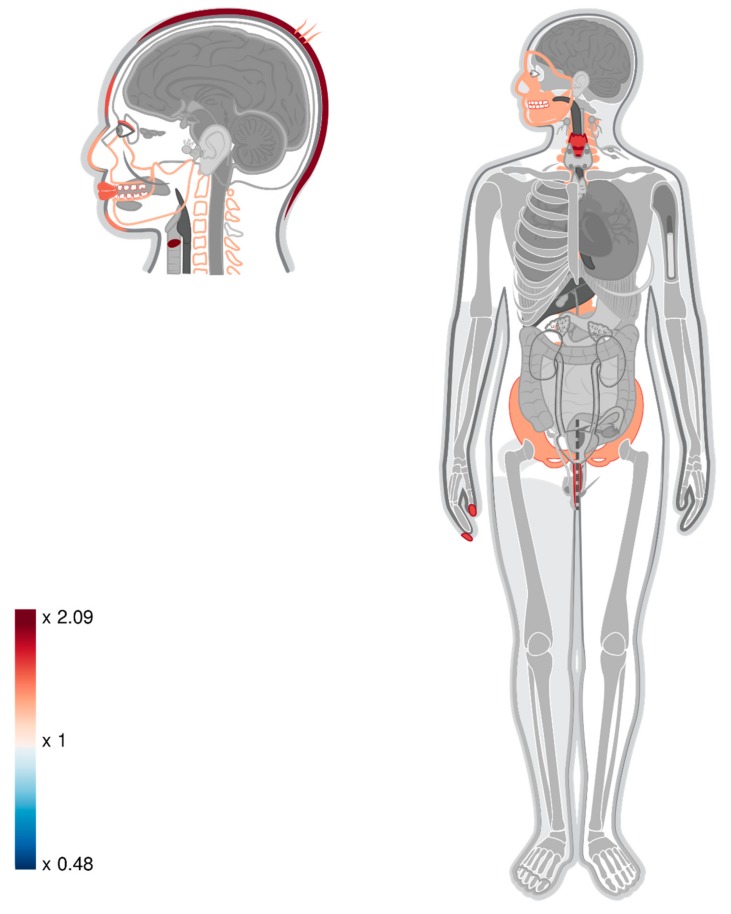
A heat map representing the level of enrichment of each anatomical part within the list of old and new DMGs based on intersection-TADs. Only body parts that are significantly enriched (FDR < 0.05) are colored. Notice the enrichment of genes connected to the chin, hair, scalp, and spinal-column.

In order to identify body parts that are expected to be affected by changes in expression level of these genes, we used Gene ORGANizer [11] on the combined list of new and previously identified DMGs. We found that the previously reported enriched body parts (mostly related to the face and larynx) become even more significant, and that new body parts are now significantly enriched with the DMGs, including the chin, hair, scalp, and spinal-column (Figure 2, Table 1, Supplementary Figure S1).

## 4. Discussion

In this study, we used 3D genomic organization data in order to seek possible impacts of non-genic DMRs on gene expression in modern humans. We first identified TADs that are likely to be present in bone tissue, and showed that MH-derived DMRs tend to reside in TADs, and to be closer to their anchor points. We then redefined the definition of a distance between a DMR and a gene, and were able to link ~70 of the non-genic DMRs to genes. In a previous work we showed that genic-DMRs are linked to genes that tend to affect vocal and facial anatomy, and suggested that these genes might have played a role in forming the phenotypic changes in these organs throughout human evolution [13,14]. Our current results reinforce these previous results, and identify additional body parts (the chin, spinal column, hair, and scalp) that are associated with differential methylation between modern and archaic humans. This work sets a proof of concept that using 3D genomic information may enhance our ability to identify regulatory changes.

**Figure 3:**
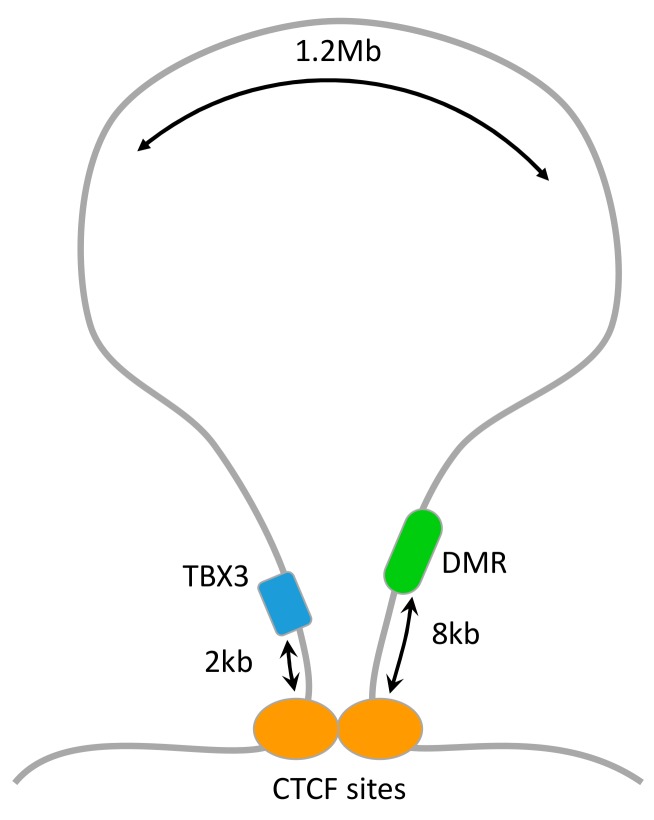
An illustration of TBX3 and its spatially associated DMR. The black arrows represent the distance between the DMR and a ChIA-PET validated CTCF binding site and TBX3 and a ChIA-PET validated CTCF binding site [23]. The linear distance between TBX3 and the DMR is 1.2 Mb.

Our redefinition of a distance between a DMR and a gene uses information on TADs in two ways. First, we use a relatively large linear distance (50 kb) between a DMR and a gene in order to offer a link between them, as we do it only if we know that the two reside within the same TAD. Second, we define a spatial distance connecting a DMR and a gene at two ends of a TAD. Seven and four of the ngDMRs in intersection TAD and union TAD analyses, respectively, were found to be close to a gene only based on spatial distance. For example, TBX3, a gene responsible for the ulnar mammary syndrome [15], lies directly close to its associated ngDMR (Figure 3).

A well-characterized difference between the modern human and the Neanderthal is the chin, which is present only in modern humans, likely as a result of face flattening [16]. In fact, the presence of a chin has long been recognized as unique to *Homo sapiens* among mammals [17]. Our results show a significant enrichment of DMGs that are known to phenotypically affect the chin, including FIG4, AXIN2, FZD2, SMARCA2, and TBX3. These genes affect the entire skeletal system, and likely affect the chin by controlling the level of facial protrusion [18,19,20,21].

Another known phenotypic difference between modern humans and Neanderthals is in the spinal column. It was suggested that Neanderthals had a natural lumbar kyphosis (i.e., their back was bent), suffered from less degenerative changes in their spinal column, and presumably did not suffer from lower back pain [22].

As ancient methylation is reconstructed in archaic bones, our DMGs are expected to be enriched within the skeletal system. It is therefore of note that we have identified scalp and hair as tissues that might be affected by differential methylation. As these tissues are not preserved in the fossil record, these predictions are difficult to test.

One important weakness of our analyses is the absence of TAD maps in human bone. Although highly conserved, the consensus TAD maps we obtained using four different cell lines represent only a fraction of the actual bone TADs. Our analysis therefore represents an underestimate of the potential 3D contacts between DMRs and genes. Once Hi-C data will become available in human bone, a more complete picture can be obtained, and we will likely be able to assign an even larger number of DMRs to function.

## Figures and Tables

**Table 1:** Anatomical parts significantly enriched in any of the analyses. Previous results that did not use topological information are compared to the results obtained here. FDR values in the current analyses decreased, and new anatomical parts became significant: spinal column, chin, hair, and scalp.

Organ Name	Results Reported in Gokhman, et al., (2017) [11]					Intersection-TAD					Union-TAD				
	Enrichment Ratio	Observed	Expected	*p*-Value	FDR	Enrichment Ratio	Observed	Expected	*p*-Value	FDR	Enrichment Ratio	Observed	Expected	*p*-Value	FDR
face	1.291	80	61.975	0	0.02	1.315	89	67.656	0	0.006	1.315	89	67.656	0	0.006
forehead	1.568	48	30.61	0	0.017	1.586	53	33.416	0	0.006	1.586	53	33.416	0	0.006
vocal cords	2.109	25	11.852	0	0.017	2.087	27	12.939	0	0.006	2.009	26	12.939	0	0.011
lips	1.465	46	31.4	0.002	0.046	1.517	52	34.278	0	0.012	1.546	53	34.278	0	0.008
eyelid	1.386	52	37.515	0.003	0.046	1.416	58	40.954	0.001	0.021	1.416	58	40.954	0.001	0.023
teeth	1.528	40	26.178	0.002	0.046	1.54	44	28.578	0.001	0.021	1.505	43	28.578	0.002	0.033
hair	1.316	49	37.24	0.012	0.1	1.377	56	40.654	0.002	0.029	1.353	55	40.654	0.004	0.04
maxilla	1.301	65	49.951	0.003	0.046	1.302	71	54.53	0.002	0.029	1.284	70	54.53	0.003	0.04
jaws	1.291	65	50.329	0.004	0.046	1.292	71	54.943	0.002	0.029	1.274	70	54.943	0.004	0.04
nails	1.602	30	18.723	0.004	0.048	1.615	33	20.439	0.002	0.029	1.566	32	20.439	0.005	0.04
larynx	1.682	29	17.246	0.002	0.046	1.647	31	18.827	0.003	0.029	1.593	30	18.827	0.005	0.04
pelvis	1.335	58	43.458	0.003	0.046	1.328	63	47.442	0.003	0.029	1.349	64	47.442	0.002	0.033
spinal column	1.296	57	43.974	0.008	0.069	1.312	63	48.005	0.004	0.032	1.333	64	48.005	0.002	0.033
mandible	1.276	64	50.157	0.006	0.055	1.278	70	54.755	0.004	0.032	1.26	69	54.755	0.006	0.049
nose	1.325	55	41.5	0.006	0.055	1.324	60	45.304	0.004	0.032	1.346	61	45.304	0.002	0.033
scalp	1.811	14	7.73	0.02	0.134	2.015	17	8.438	0.004	0.032	2.015	17	8.438	0.004	0.04
urethra	1.365	22	16.112	0.067	0.22	1.592	28	17.589	0.007	0.049	1.535	27	17.589	0.012	0.08

## Supplementary Materials

The following are available online at https://www.mdpi.com/2073-4409/9/1/48/s1, Figure S1: A heat map representing the level of enrichment of each anatomical part within the list of old and new DMGs based on union-TADs, Table S1: Number and median length of identified TADs in four different cell types, Table S2: MH-derived DMRs tend to reside inside TADs, Table S3: MH-derived DMRs tend to reside close to TAD boundaries.
