# Supplementary material for: Predicted Archaic 3D Genome Organization Reveals Genes Related to Head and Spinal Cord Separating Modern from Archaic Humans
Source: Cells. 2019 Dec 24;9(1):48. doi: 10.3390/cells9010048 (PMC7017363; doi:10.3390/cells9010048)
Supplement: Supplementary file 1 [file cells-09-00048-s001.pdf]

Supplementary Materials

Tables

Table S1. Number and median length of identified TADs in four different cell types. Data are taken from [6].

Cell Type	# TADs	Median TAD Length
HMEC	4096	230,000
HUVEC	4173	270,000
IMR90	7680	280,000
NHEK	6015	250,000

Table S2. MH-derived DMRs tend to reside inside TADs.

	Intersection-TADs	Union-TADs
Observed fraction of DMRs in TADs	0.43986	0.48225
# of MH-DMRs inside TAD	384	421
Total # of MH-DMRs	873	
Expected fraction of DMRs in TADs (from simulations)	0.31897	0.35818
p-value of Monte Carlo simulation	$p < 0.005$	$p < 0.005$
P-value of χ^2-test	$p = 1.8 \cdot 10^{-14}$	$p = 2.1 \cdot 10^{-14}$

Table S3. MH-derived DMRs tend to reside close to TAD boundaries.

(Only DMRs Inside TADs)	Intersection-TADs	Union-TADs
Observed fraction of DMRs within 50 kb of TAD boundary	0.73177	0.66983
# MH-DMRs –TAD end < 50 kb	281	282
Total # of MH-DMRs inside TADs	384	421
Expected fraction of DMRs within 50 kb of TAD boundary (from simulations)	0.35229	0.30705
p-value of Monte Carlo simulation	$P < 10^{-4}$	$P < 10^{-4}$
p-value χ^2-test	$P = 1.2 \cdot 10^{-54}$	$\mathrm{P} = 1.4 \cdot 10^{-58}$

Figure

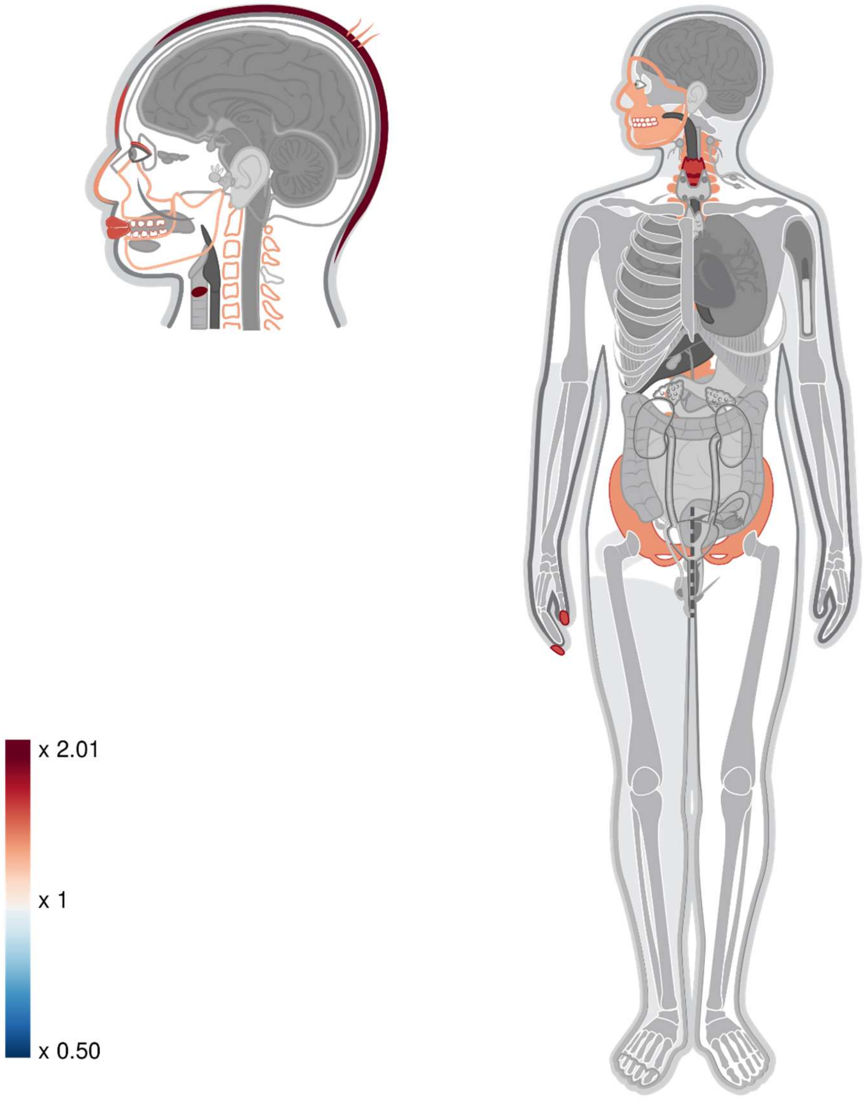

Figure S1. A heat map representing the level of enrichment of each anatomical part within the list of old and new DMGs based on union-TADs. Only body parts that are significantly enriched (FDR < 0.05) are colored.
